# Adverse Drug Reaction-Related Hospital Admissions and Adverse Drug Events and Their Association with Short- and Long-Term Health Outcomes in Older Adults

**DOI:** 10.3390/geriatrics11010011

**Published:** 2026-01-20

**Authors:** Juliane Frydenlund, David J. Williams, Frank Moriarty, Emma Wallace, Ciara Kirke, Kathleen Bennett, Caitriona Cahir

**Affiliations:** 1 Data Science Centre, School of Population Health, RCSI University of Medicine and Health Science, D02 YN77 Dublin, Ireland; julianefrydenlund@rcsi.ie (J.F.); kathleenebennett@rcsi.ie (K.B.); 2 Department of Geriatric and Stroke Medicine, RCSI University of Medicine and Health Sciences, D09 YD60 Dublin, Ireland; davidwilliams@rcsi.ie; 3 School of Pharmacy and Biomolecular Sciences, RCSI University of Medicine and Health Sciences, D02 YN77 Dublin, Ireland; frankmoriarty@rcsi.ie; 4 Department of General Practice, School of Medicine, University College Cork, T12 R229 Cork, Ireland; ewallace@ucc.ie; 5 National Quality and Patient Safety at Health Service Executive, D08 W2A8 Dublin, Ireland; ciara.kirke@hse.ie

**Keywords:** adverse drug reactions (ADR), adverse drug events (ADE), health related quality of life (HRQOL), older populations

## Abstract

**Background**: This study examined whether adverse drug reaction (ADR)-related hospital admissions or adverse drug events (ADE) in primary care are associated with changes in health-related quality of life (HRQOL), functional decline, and A&E visits, over time, in two separate prospective cohort studies of older adults in Ireland. **Methods**: The Adverse Drug reactions in an Ageing PopulaTion (ADAPT) (Study 1: N = 230) and the Centre for Primary Care Research (CPCR) (Study 2: N = 605) prospective cohorts were used. Participants completed health outcome questionnaires at baseline and again at 3 months (Study 1) and at 24 months (Study 2). ADR-related admissions and ADEs were assessed at baseline. Multivariable linear, logistic, and ordinal logistic regressions were used to examine associations between ADR-related admissions/ADEs and changes in HRQOL (EQ-5D-5L/3L), functional decline, and A&E visits, adjusting for age, sex, comorbidity, and polypharmacy. **Results**: In Study 1 (ADAPT cohort), frailty increased and A&E visits decreased over 3 months in both ADR/non-ADR groups (*p* < 0.01). In Study 2 (CPCR cohort), HRQOL decreased, and functional decline and A&E visits increased for both ADE/non-ADE groups over 24 months (*p* < 0.05). Individuals with ADEs had lower HRQOL and greater functional decline at both time points (*p* < 0.001). However, experiencing an ADR or an ADE was not significantly associated with changes in HRQOL, functional decline, or A&E visits over time, after adjustments. **Conclusions**: There were no substantial differences in the short-term healthcare burden of ADRs, while ADEs had poorer long-term outcomes.

## 1. Background

Adverse drug reactions (ADR) and adverse drug events (ADE) are unintended, harmful responses to a medication and can vary in severity, from mild side effects to severe or life-threatening conditions [1,2]. An ADR is defined as “an appreciably harmful or unpleasant reaction resulting from an intervention relating to the use of a medicinal product” [1]. An ADE is any untoward medical occurrence that may present during treatment with a medicinal product but is not necessarily caused by the drug [3]. ADRs can be thought of as a subset of ADEs, where a causal link to a medicine has been established [4,5]. ADRs and ADEs can occur due to various factors, including the drug’s pharmacological properties, interactions with other medications, patient-specific factors (such as age, genetics, or underlying health conditions), and improper use of the medication [4,6].

Older people have an increased risk of experiencing ADRs and ADEs due to greater morbidity and medication utilisation and a variety of physiological changes affecting the pharmacokinetics and pharmacodynamics of medications [7]. A systematic review and meta-analysis has estimated the overall percentage of hospital admissions in older adults due to ADRs to be 8.7% [8]. Studies have also indicated that 11.75–18% of hospital admissions in older people are due to ADEs [9]. A number of risk factors have also been identified as being associated with ADRs and ADEs in older populations, including being female, advanced age, increased disease burden, number of medications, and polypharmacy [10].

While studies have explored the prevalence and risk factors of ADRs and ADEs in older patients, few studies have investigated the short- and longer-term health outcomes associated with these events across both primary and secondary care settings. There is evidence that ADRs and ADEs are associated with morbidity, mortality, and increased costs [11,12,13]. In secondary care, studies have found that those admitted with an ADR have longer hospital stays and higher rates of readmission than those without ADRs [13]. However, the impact of ADRs and ADEs may extend beyond acute hospital-based outcomes. In both primary and secondary care, these events may lead to additional physical and psychological morbidity in older people, including reduced health-related quality of life (HRQOL), functional impairment, difficulty engaging in daily activities, and increased anxiety, depression, or stress, or, indeed, further hospital visits [14]. Social engagement may decline due to reduced physical, functional, and emotional well-being, affecting relationships and support networks [15].

Further research is required to understand the short- and long-term healthcare burden of experiencing an ADR or an ADE in older people in primary and secondary care settings. This knowledge will help healthcare providers to monitor and provide additional supports to individual patients as needed to prevent further morbidity or gradual health deterioration over time. The aim of this study is to examine whether (i) experiencing an ADR-related hospital admission (Study 1) or (ii) an ADE in primary care (Study 2) is associated with changes in health outcomes over time, including HRQOL, functional decline, and A&E visits, in two separate prospective cohort studies of older adults in Ireland.

## 2. Methods

The STROBE guidelines were utilised for reporting the observational study [16]. In this study, two separate sources of data were used: the Adverse Drug reactions in an Ageing PopulaTion (ADAPT) cohort (Study 1) [17,18] and the Centre for Primary Care Research (CPCR) cohort (Study 2) [19].

### 2.1. Study 1: The ADAPT Cohort

#### 2.1.1. Setting and Study Design

The ADAPT cohort (N = 798) is a cross-sectional study of prevalence and risk factors for ADRs in all community-based adults aged ≥65 years admitted acutely to a large tertiary referral hospital in Ireland over an eight-month period (November 2016–June 2017) and a prospective cohort study of patient-reported health outcomes associated with ADR-related hospital admissions. A subset of those with and without an ADR (non-ADR) from the initial cross-sectional study were invited to take part in the prospective cohort study of patient-reported outcomes. In total, 230 patients (93 with an ADR-related hospital admission; 137 non-ADR-related hospital admission) completed a baseline questionnaire of patient-reported health outcomes at baseline T1a (2016–2017), and a follow-up questionnaire three months post-discharge at timepoint T2a (2017) (short-term). A protocol and detailed description of the ADAPT cohort has been previously published [17,18].

#### 2.1.2. ADR-Related Hospital Admissions

In the ADAPT cohort, ADR-related admissions were determined at baseline (T1a) using a multifaceted review of each hospital admission to assess the likelihood of the ADR being a reason for admission (cause of admission or contributing to admission) in the context of the patient’s medication, clinical conditions, medical history, comorbidities, and investigations, and using validated algorithms and decision aids. A detailed analysis of the prevalence and nature of the ADR-related hospital admissions (causality, severity, preventability) within the ADAPT cohort has been published previously [17,18,20].

#### 2.1.3. Short-Term Health Outcomes

Short-term (3-month) health outcomes in the ADAPT cohort included HRQOL, functional decline, and A&E visits and were measured at both baseline (T1a) and at the 3-month follow-up (T2a). HRQOL was measured using the EQ-5D-5L score [21]. EQ-5D-5L health states were converted into a single utility value for each patient based on the Irish population value set [22]. Functional decline was measured using the Groningen Frailty Index (GFI) and includes four domains: (1) physical (mobility functions, multiple health problems, physical fatigue, vision, hearing); (2) cognitive (cognitive dysfunction); (3) social (emotional isolation); and (4) psychological (depressed mood and feelings of anxiety). A score of ≥4 indicates functional decline and frailty [23]. The number of A&E visits in the previous 3 months was measured at baseline (T1a) and again at the 3-month follow-up (T2a) per participant self-report.

#### 2.1.4. Covariates

Age, sex, comorbidity, and polypharmacy were included as covariates and were measured at baseline. Comorbidity burden was measured using the Charlson Comorbidity Index [24]. Polypharmacy was defined as ≥5 drug classes (ATC level 3), and significant polypharmacy as ≥10 [25].

### 2.2. Study 2: The CPCR Cohort

#### 2.2.1. Setting and Study Design

The CPCR cohort (N = 904) is a prospective cohort study of ADEs, risk factors for ADEs, and health outcomes in patients aged ≥70 years from 15 primary care practices in Ireland. In total, 605 patients (465 with an ADE; 126 non-ADE (14 missing)) completed a baseline questionnaire of patient-reported health outcomes at timepoint T1b (2010–2011) and a follow-up questionnaire 24 months later at timepoint T2b (2012–2013) (longer-term). A detailed description of the CPCR cohort has been previously published [19,26].

#### 2.2.2. ADEs

In the CPCR cohort, ADEs were determined at baseline (T1b) through patient interviews and review of GP medical records in the previous 6 months. The ADEs determined in the CPCR cohort were primarily mild, and few resulted in hospital admission. All patient-reported ADEs were independently reviewed by two academic GPs, who rated the likelihood of each patient-reported ADE being a true ADE on a Likert 6-point scale (1 = no confidence to 6 = certain). Only ADEs where both reviewers rated the ADE as likely (≥50% likelihood; score ≥ 4) were included. A detailed analysis of the prevalence and nature of the ADEs within the CPCR cohort has been published previously [19].

#### 2.2.3. Long-Term Health Outcomes

Long-term (24-month) health outcomes in the CPCR cohort, similarly included HRQOL, functional decline, and A&E visits, and were measured at both baseline (T1b) and at the 24-month follow-up (T2b). HRQOL was measured using EQ-5D-3L, and the utilities were converted based on the UK population value set (Irish population set not available) using the time trade-off valuation technique [27]. Functional decline was measured using the Vulnerable Elders Survey (VES). The VES includes 13 items relating to patient age, self-rated health, ability to perform six physical tasks (e.g., writing or handing small objects, walking a quarter of a mile, lifting), and 5 items relating to function (e.g., bathing, managing finances, light housework). A score of ≥3 indicates a higher risk of experiencing future functional decline [28]. The number of A&E visits in the previous 6 months was measured at baseline (T1b) and during the 24-month follow-up period (T2b) per participant’s GP electronic medical record.

#### 2.2.4. Covariates

Covariates included age, sex, comorbidity, and polypharmacy, measured at baseline, using the same methodology as the ADAPT cohort.

### 2.3. Data Analysis

#### 2.3.1. Study 1 and 2 Population Characteristics

Descriptive statistics including medians (inter-quartile range, IQR) and proportions, were calculated for all three health outcomes (HRQOL, functional decline, A&E visits) and covariates in Study 1 (ADAPT cohort) and Study 2 (CPCR cohort). Differences in covariates were compared between those experiencing ADR-related hospital admissions versus non-ADR-related admissions in Study 1 (ADAPT cohort) and ADEs versus non-ADEs in Study 2 (CPCR cohort) using chi-square tests for categorical variables and non-parametric Wilcoxon–Mann–Whitney tests for continuous variables at baseline (T1a and T1b).

#### 2.3.2. Changes in Health Outcomes Between Baseline and Follow-Up and Differences Between ADR/Non-ADR Groups (Study 1) and ADE/Non-ADE Groups (Study 2)

Changes in HRQOL between baseline and follow-up were examined for patients with an ADR-related hospital admission and those without (Study 1 T1a vs. T2a) and patients with an ADE and those without (Study 2 T1b vs. T2b) using Wilcoxon matched-pairs signed-rank tests. Changes in functioning and number of A&E visits between baseline and follow-up were examined for patients with an ADR-related hospital admission and those without (Study 1 T1a vs. T2a) and patients with an ADE and those without (Study 2 T1b vs. T2b) using McNemar tests. For the number of A&E visits, the McNemar tests compared 1 A&E visit to no visit and ≥2 A&E visits to 1 A&E visit and to no visit, respectively. Differences in HRQOL, functional decline, and number of A&E visits between patients with an ADR-related hospital admission versus those without (Study 1 T1a vs. T2a) and patients with an ADE versus those without (Study 2 T1b vs. T2b) were assessed at both baseline and follow-up, using Wilcoxon–Mann–Whitney tests for HRQOL and chi-square tests for functional decline and A&E visits.

#### 2.3.3. ADR (Study 1) and ADE (Study 2) Exposure and Change in HRQOL, Functional Decline, and Number of A&E Visits

Multivariable linear, logistic, and ordinal logistic regressions were conducted separately for Study 1 and Study 2, respectively, to examine the change in HRQOL (EQ-5D-5L and EQ-5D-3L; continuous), functional decline (GFI and VES; binary), and number of A&E visits (ordinal data) between baseline and follow-up associated with ADR-related hospital admission compared to non-ADR-related admission (Study 1 T2a-T1a) in the ADAPT cohort and ADEs compared to non-ADEs (Study 2 T2b-T1b) in the CPCR cohort. The models were adjusted for age, gender, comorbidity, and polypharmacy. The multivariable logistic regression model examining the change in functional decline (VES) in Study 2 (CPCR cohort) was not adjusted for age, as age is included as part of the VES score. All multivariable linear regression models used robust standard errors. In Study 2 (CPCR cohort), all multivariable regression models were also adjusted for clustering of individuals within the 15 general practices. The data was analysed using Stata Version 18.0 (StataCorp, College Station, TX, USA).

## 3. Results

### 3.1. Study 1 and 2 Population Characteristics

Table 1 presents the baseline characteristics of the Study 1 (ADAPT T1a) cohort by ADR/non-ADR and the Study 2 (CPCR T1b) cohort by ADE/non-ADE patients.

There were no significant differences in participant characteristics between the ADR and non-ADR groups in the Study 1 ADAPT cohort. In Study 2, the CPCR cohort, there was a higher proportion of significant polypharmacy and morbidity in those with an ADE compared to those without an ADE (*p* < 0.001).

### 3.2. Changes in Health Outcomes Between Baseline and Follow-Up and Differences Between ADR/Non-ADR Groups (Study 1) and ADE/Non-ADE Groups (Study 2)

#### 3.2.1. Study 1: The ADAPT Cohort

In the ADAPT cohort at baseline (T1a), the overall median EQ-5D-5L was 0.66 (IQR: 0.34–0.91), 164 (71%) patients were determined to be frail, and 46 (20%) patients had two or more A&E visits in the previous 3 months.

Table 2 presents HRQOL, functional decline, and number of A&E visits at baseline (T1a) and at follow-up (T2a) for ADR and non-ADR patients. The proportion of patients who were frail significantly increased between baseline (T1a) and follow-up (T2a) for both those with an ADR-related hospital admission (68% to 85%; *p* = 0.003) and those without an ADR-related admission (74% to 85%; *p* = 0.005). There was also a significant decrease in the number of A&E visits (≥2 visits vs. none) between baseline (T1a) and follow-up (T2a) for those with an ADR-related hospital admission (*p* = 0.003) and those without (*p* < 0.001).

#### 3.2.2. Study 2: The CPCR Cohort

In the CPCR cohort at baseline (T1b), the overall median EQ-5D-3L was 0.80 (IQR: 0.69–1.00), 197 (33%) had a high risk of experiencing future functional decline, and 11 (2%) had two or more A&E visits in the previous 6 months. Table 3 presents HRQOL, functional decline, and number of A&E visits at baseline (T1b) and at follow-up (T2b) for ADE and non-ADE patients. There was a significant decrease in HRQOL between baseline (T1b) and follow-up (T2b) for both ADE (*p* = 0.001) and non-ADE (*p* = 0.044) patients. The proportion of patients at risk of functional decline also significantly increased between baseline (T1b) and follow-up (T2b) for both ADE (35% to 40%; *p* = 0.020) and non-ADE (17% to 25%; *p* = 0.039) patients. The number of A&E visits between baseline (T1b) and follow-up (T2b) significantly increased for those with an ADE and those without (*p* < 0.001). Patients with an ADE had a significantly lower HRQOL at baseline (T1b; 0.74 vs. 0.81; *p* < 0.001) and at follow-up (T2b; 0.73 vs. 0.80; *p* < 0.001) compared to those without an ADE. A higher proportion of patients with an ADE were frail at baseline (T1b; 35% vs. 17%; *p* < 0.001) and at follow-up (T2b; 40% vs. 25%; *p* < 0.001) compared to those without an ADE.

### 3.3. ADR (Study 1) and ADE (Study 2) Exposure and Change in HRQOL, Functional Decline, and Number of A&E Visits

#### 3.3.1. Study 1: The ADAPT Cohort

Table 4 presents the change in HRQOL, functional decline, and number of A&E visits, respectively, between baseline and follow-up (T2a-T1a) associated with ADR-related hospital admission compared to non-ADR-related admission. In general, there was no significant difference in the change in HRQOL, functional decline, and number of A&E visits between baseline and follow-up for those with an ADR-related hospital admission compared to those without (non-ADR). Male patients were twice as likely to experience a change (adjusted OR 2.22, 95% CI: 1.14, 4.32) in functional decline between baseline and follow-up compared to females after adjusting for covariates.

#### 3.3.2. Study 2: The CPCR Cohort

Table 5 presents the change in HRQOL, functional decline, and number of A&E visits, respectively, between baseline and follow-up (T2b-T1b) for patients with an ADE compared to those without an ADE. In general, there was no significant difference in the change in HRQOL, functional decline, and number of A&E visits between baseline and follow-up for those with an ADE compared to those without (non-ADE).

## 4. Discussion

In these two studies we examined the association between experiencing an ADR-related hospital admission (study 1) or an ADE (study 2) and short- and long-term health outcomes, respectively, including HRQOL, functional decline, and A&E visits in older adults. In Study 1 (ADAPT cohort), the proportion of patients classified as frail increased significantly, while the number of A&E visits decreased between hospital admission and 3 months post-discharge in both patients with an ADR-related hospital admission and without. There were no significant differences in health outcomes between the two groups at baseline (hospital admission) or at 3-month follow-up. Similarly, changes in HRQOL, functional status, and A&E visits over the 3-month period did not differ significantly between patients with and without an ADR-related hospital admission.

In Study 2 (CPCR cohort), HRQOL declined significantly over 24 months, while the proportion of patients at risk of functional decline and the number of A&E visits increased for both those with an ADE and those without an ADE over the 24-month period. Patients with an ADE had significantly lower HRQOL and a higher risk of functional decline than those without an ADE at baseline and again at 24 months. However, the magnitude of change in HRQOL, functional status, and A&E visits over the 24-month period did not differ significantly between patients with and without an ADE.

There is limited research examining the impact of ADRs and ADEs on HRQOL in older populations. A retrospective cross-sectional study from the Netherlands reported that patients who experienced ADRs due to levothyroxine had a significant decrease in HRQOL after the event [29]. However, the study involved a younger population and the impact of ADRs in relation to a change in packaging, and lacked a non-ADR comparison group. Another study of psychiatric outpatients in New Delhi found that ADRs associated with antipsychotic use were related to lower HRQOL at baseline and 3-month follow-up in the physical and psychological domains but not the social and environmental domains [30]. In the current study, the ADAPT cohort comprised older, frail patients with multiple comorbidities, prescribed multiple medications, who had substantially lower HRQOL than both the primary care CPCR cohort and the general Irish population [19,22]. Although mean HRQOL improved slightly between hospital admission and 3 months post-discharge (by 0.06) among patients with an ADR-related hospital admission, the change was not statistically significant, largely due to wide variability in utility scores within the cohort. The national EQ-5D-5L Irish value set assigns relatively high decrements to anxiety/depression and pain/discomfort compared with other countries’ value sets, which may have influenced these findings; in this cohort, mobility, self-care, and usual activities showed little change post-discharge, whereas anxiety/depression and pain/discomfort improved [22,31].

In the primary care setting (Study 2 CPCR cohort), patients who experienced an ADE had a clinically meaningful lower HRQOL (minimally important difference (MID) = 0.07), compared with those without an ADE, both at baseline and 24 months later [31]. Non-serious symptomatic ADEs such as headaches, itchiness, or muscle pain can have a substantial impact on patients’ HRQOL [14]. However, their impact is often underestimated by clinicians, who may focus more on severe ADEs that directly influence morbidity or mortality. Qualitative research has also shown that patients commonly report medication effects (e.g., rash, muscle pain) as severe in lived experience, even when clinicians might label them as mild [32]. In contrast to ADR-related hospital admission, where acute symptoms are typically treated promptly and HRQOL may improve [33], less severe ADEs in the primary care setting may remain unreported, contributing to persistently poorer HRQOL. Prior research indicates that 30% to 50% of patients do not report medication-related adverse effects to their healthcare provider [34].

In the ADAPT cohort, all patients experienced significant functional decline/increased frailty over time, regardless of whether they experienced an ADR. Research has shown that transitions to worse frailty states are more common than improvements, and increasing frailty can trigger a downward spiral marked by greater risks of disability, falls, hospitalisation, and death [35]. Older adults are particularly vulnerable, with evidence demonstrating a higher likelihood of functional decline and medical complications following A&E visits [36]. Male patients in ADAPT were more likely to experience functional decline over the 3 months post-discharge compared to female patients, despite some evidence suggesting that male sex may be protective against frailty, partly due to higher muscle mass [37].

It has also been argued that frailty as a geriatric syndrome may have greater clinical relevance in primary care than in acute settings, where its usefulness for risk stratification remains uncertain [38]. In the primary care cohort (CPCR), all patients similarly experienced functional decline over the 24-month period; however, those who experienced an ADE were significantly frailer than those who did not at both time points. Longitudinal data from community-dwelling older adults in five European countries support a cyclical relationship in which individuals with greater frailty are at higher risk of medication-related problems, including ADE, which may, in turn, contribute to further frailty progression [39].

In the ADAPT cohort, the number of A&E visits significantly declined in the three months following hospital discharge compared with the three months preceding admission. In contrast, within the primary care (CPCR) cohort, A&E visits increased significantly over the 24-month period for all patients. The absence of a difference in A&E utilisation between ADR/ADE and non-ADR/ADE groups in both cohorts contrasts with previous research. For example, a retrospective cohort study in France reported that approximately half of patients hospitalised for an ADR were readmitted within one year of discharge [40]. Similarly, a US hospital readmission study found a 25% increase in all-cause readmissions among older patients with ADEs compared with those without [13]. One possible explanation in the ADAPT cohort is that patients may have had more frequent A&E visits in the period immediately preceding hospital admission, which naturally decreased after treatment and discharge; different patterns might have emerged over a longer follow-up. In the CPCR cohort, the absence of differences between ADE and non-ADE groups may reflect the mild nature of most ADEs, which were unlikely to lead to additional A&E visits.

### 4.1. Limitations

This study is among the first to examine both the short- and long-term burden of ADRs and ADEs in older adults. However, there are several limitations. In Study 1, the ADAPT cohort is based on a single tertiary referral hospital and included older, frail patients with multiple comorbidities who were prescribed, on average, 10 or more medications [17,18]. In Study 2, the CPCR cohort included 15 general practices from one region of Ireland [19]. The results may not be generalisable to other healthcare settings or to the general older population. Although ADRs and ADEs were identified through clinical judgement, chart review, and the use of validated algorithms (for ADRs), some degree of misclassification remains possible. Additionally, the health outcome measures used in the two studies were similar but not identical, and consequently, the findings are presented individually for each cohort and are not directly comparable.

More broadly, EQ-5D measures have been criticised for lacking nuance and failing to capture important aspects of patients’ lived experience, suggesting that additional HRQOL instruments should be incorporated in future research [41]. While the feasibility, reliability, and validity of the GFI and VES for detecting functional deficits in older adults have been demonstrated [42,43], the sensitivity of other measures of functional decline or frailty in relation to ADRs and ADEs should also be evaluated in future studies. Finally, although both studies adjusted for a range of baseline covariates, these factors may have changed over time, and many other predictors of health outcomes in older adults were not captured. Residual and unmeasured confounding, therefore, cannot be excluded.

### 4.2. Implications

Medication-related harm in older adults, including ADRs and ADEs, has been recognised internationally as a priority area for improvement across all healthcare settings. The World Health Organization (WHO) has designated Medication Safety as the focus of its third Global Patient Safety Challenge, with a target of reducing severe, avoidable medication-related harm by 50% over five years [44]. In this study, two distinct older populations were examined: an acutely ill cohort admitted to hospital and a primary care cohort, enabling assessment of both the short- and long-term healthcare burden associated with medication-related harm.

Interestingly, no substantial difference emerged in the short-term healthcare burden between ADR-related and non-ADR acute admissions. Previous analyses of the ADAPT cohort showed that ADR-related hospitalisations were associated with only modest incremental costs during admission and over the following three months [20]. Evidence suggests that in frail, multimorbid older adults presenting acutely, even commonly used medicines may cause harm due to reduced physiological reserves and heightened vulnerability to minor side effects. Despite this, awareness of the unique burden and consequences of ADRs in this population remains limited, as do strategies to mitigate these risks [45]. Further research involving larger and more heterogeneous populations is needed to better understand the longer-term impact of ADRs and the types of ADRs on health outcomes, including progressive functional decline and increasing frailty.

In the primary care setting, a different pattern was observed. Patients who experienced ADEs had poorer long-term outcomes than those who did not, including lower HRQOL and greater frailty at baseline and follow-up. This highlights the need for improved methods or tools to support early detection of ADEs in primary care, which is a challenge given the substantial overlap between ADE symptoms, comorbid conditions, and normal ageing. Although many ADEs are mild, they can meaningfully affect patients’ HRQOL and may progress over time into more serious adverse effects requiring medical attention or hospitalisation. Enhanced patient health literacy regarding medications, alongside routine medication reviews, integration of clinical pharmacists into primary care, and adopting a more collaborative, patient-centred approach, may help older patients recognise medications as a potential cause of new symptoms and reduce the long-term impact of ADEs on health outcomes [5,46].

## 5. Conclusions

This study highlights the need for further research to better understand the impact of ADRs and ADEs on older patients’ lives, particularly in relation to HRQOL, frailty, and overall well-being. Advancing this evidence-base will not only provide a greater understanding of the broader impact of medication-related harm on older people but will also inform the development of support and interventions to prevent, detect, and manage adverse effects of medication, ultimately improving the healthcare and well-being of older patients.

## Figures and Tables

**Table 1:** Baseline (T1a and T1b) characteristics of the Study 1 ADAPT and Study 2 CPCR cohorts by ADR/non-ADR and ADE/non-ADE patients.

		ADAPT N = 230 (Baseline T1a)			CPCR N = 605 (Baseline T1B)		
		ADR (N = 93)	Non-ADR (N = 137)	*p*-Value	ADE (N = 465)	Non-ADE (N = 126)	*p*-Value
Male, N (%)		52 (56%)	64 (47%)	0.171	212 (46%)	65 (52%)	0.232
Age, Median (IQR)		79 (75–86)	80 (74–86)	0.544	79 (75–82)	78 (75–82)	0.142
CCI, N (%)	0	16 (17%)	25 (18%)	0.805	212 (46%)	92 (73%)	*p* < 0.001
	1–2	37 (40%)	59 (43%)		218 (47%)	28 (22%)	
	≥3	40 (43%)	53 (39%)		35 (8%)	6 (5%)	
Polypharmacy, N (%)	≤4	12 (13%)	18 (13%)	0.448	65 (14%)	56 (44%)	*p* < 0.001
	5–9	41 (44%)	71 (52%)		231 (50%)	55 (44%)	
	≥10	40 (43%)	48 (35%)		169 (36%)	15 (12%)	

Abbreviations: IQR: inter-quartile range, CCI (Charlson Comorbidity Index); 14 missing in the CPCR cohort due to no registration on ADE/non-ADR.

**Table 2:** Study 1 health outcomes (HRQOL, functional decline, A&E visits) at baseline (T1a) and at follow-up (T2a) for ADR/non-ADR patients in the ADAPT cohort.

ADAPT	ADR			Non-ADR			ADR vs. Non-ADR	
	Baseline T1a (N = 93)	Follow-Up T2a (N = 93)	*p*-Value	Baseline T1a (N = 137)	Follow-Up T2a (N = 137)	*p*-Value	Baseline T1a (*p*-Value)	Follow-Up T2a (*p*-Value)
**HRQOL**	Median (IQR)	Median (IQR)		Median (IQR)	Median (IQR)			
**EQ-5D-5L**	0.65 (0.34–0.91)	0.71 (0.37–0.88)	0.580	0.66 (0.34–0.91)	0.69 (0.32–0.88)	0.848	0.823	0.937
**Functional decline**	N (%)	N (%)		N (%)	N (%)			
**GFI < 4 (non-frail)**	30 (32%)	14 (15%)		36 (26%)	20 (15%)			
**GFI ≥ 4 (frail)**	63 (68%)	79 (85%)	0.003	101 (74%)	117 (85%)	0.005	0.325	0.924
**A&E visits**								
**0 A&E visit**	30 (33%)	48 (52%)	0.233 a	39 (30%)	81 (59%)	0.371 a		
**1 A&E visit**	37 (41%)	29 (31%)	0.406 b	70 (53%)	28 (20%)	0.484 b		
**≥2 A&E visits**	23 (26%)	16 (17%)	0.003 c	23 (17%)	28 (20%)	*p* < 0.001 c	0.171	0.179

Wilcoxon matched-pairs signed-rank tests were used to measure differences in EQ-5D-5L between baseline (T1a) and follow-up (T2a) for both ADR and non-ADR groups. McNemar tests were used to measure differences in functional decline and A&E visits between baseline (T1a) and follow-up (T2a) for both ADR and non-ADR groups. The number of A&E visits was compared as 1 visit vs. no visit (a), 2 visits vs. 1 visit (b), and 2 visits vs. no visits (c). Differences in HRQOL between ADR and non-ADR groups were assessed at baseline (T1a) and follow up (T2a) using Wilcoxon–Mann–Whitney tests. Differences in functional decline and number of A&E visits between ADR and non-ADR groups were assessed at baseline (T1a) and follow-up (T2a) using chi-square tests. Missing: 1 patient’s EQ-5D-5L(T1a), 9 patients’ EQ-5D-5L (T2a), 8 patients’ A&E visits (T1a).

**Table 3:** Study 2 health outcomes (HRQOL, functional decline, A&E visits) at baseline (T1b) and at follow-up (T2b) for ADE/non-ADE patients in the CPCR cohort.

CPCR	ADE			Non-ADE			ADE vs. Non-ADE	
	Baseline T1b (N = 465)	Follow-Up T2b (N = 465)	*p*-Value	Baseline T1b (N = 126)	Follow-Up T2b (N = 126)	*p*-Value	Baseline T1b (*p*-Value)	Follow-Up T2b (*p*-Value)
**HRQOL**	Median (IQR)	Median (IQR)		Median (IQR)	Median (IQR)			
**EQ-5D-3L**	0.74 (0.69–0.85)	0.73 (0.66–0.85)	0.001	0.81 (0.76–1.00)	0.80 (0.73–1.00)	0.044	*p* < 0.001	*p* < 0.001
**Functional decline**	N (%)	N (%)		N (%)	N (%)			
**VES < 3 (non-frail)**	300 (65%)	277 (60%)		104 (83%)	95 (75%)			
**VES ≥ 3 (frail)**	165 (35%)	188 (40%)	0.020	22 (17%)	31 (25%)	0.039	*p* < 0.001	*p* < 0.001
**A&E visits**								
**0 A&E visits**	427 (92%)	375 (81%)	*p* < 0.001 a	122 (97%)	105 (84%)	*p* < 0.001 a		
**1 A&E visit**	27 (6%)	75 (16%)	*p* < 0.001 b	4 (3%)	14 (11%)	*p* < 0.001 b		
**≥2 A&E visits**	11 (2%)	14 (3%)	*p* < 0.001 c	0 (0%)	6 (5%)	*p* < 0.001 c	0.102	0.266

Wilcoxon matched-pairs signed-rank tests were used to measure differences in EQ-5D-3L between baseline (T1b) and follow-up (T2b) for both ADE and non-ADE groups. McNemar tests were used to measure differences in functional decline and A&E visits between baseline (T1b) and follow-up (T2b) for both ADE and non-ADE groups. The number of A&E visits was compared as 1 visit vs. no visits (a), 2 visits vs. 1 visit (b), and 2 visits vs. no visits (c). Differences in HRQOL between ADE and non-ADE groups were assessed at baseline (T1b) and follow-up (T2b) using Wilcoxon–Mann–Whitney tests. Differences in functional decline and number of A&E visits between ADE and non-ADE groups were assessed at baseline (T1b) and follow-up (T2b) using chi-square tests. Missing: 14 patients’ A&E visits (T2b).

**Table 4:** Study 1 multivariate unadjusted and adjusted co-efficients and odds ratios (OR) for the change in health outcomes (HRQOL, functional impairment, A&E visits) between baseline and follow-up (T2a-T1a) for patients with ADR-related hospital admissions compared to those without (non-ADR).

ADAPT	EQ-5D-5L (Follow-Up T2a- Baseline T1a) (N = 220)		Functional Decline (GFI) (Follow-Up T2a- Baseline T1a) (N = 230)		No. of A&E Visits (Follow-Up T2a- Baseline T1a) (N = 222)	
	Unadjusted Univariate Coef. (95% CI)	Adjusted Coef. (95% CI)	Unadjusted Univariate OR (95% CI)	Adjusted OR (95% CI)	Unadjusted Univariate OR (95% CI)	Adjusted OR (95% CI)
**ADR (vs. non-ADR)**	−0.05 (−0.14; 0.04)	−0.06 (−0.15; 0.03)	1.41 (0.78; 2.56)	1.55 (0.81; 2.96)	1.04 (0.65; 1.68)	1.06 (0.64; 1.76)
**Age**	−0.00 (−0.01; 0.00)	−0.00 (−0.01; 0.00)	0.99 (0.95; 1.02)	1.00 (0.96; 1.04)	0.99 (0.96; 1.02)	0.99 (0.96; 1.02)
**Male (vs. female)**	0.04 (−0.05; 0.13)	0.06 (−0.05; 0.16)	2.04 (1.11; 3.74)	2.22 (1.14; 4.32) *	0.79 (0.49; 1.26)	0.88 (0.52; 1.50)
**CCI (vs. none)**						
1–2	0.09 (−0.02; 0.20)	0.06 (−0.07; 0.20)	0.71 (0.33; 1.52)	0.79 (0.32; 1.95)	1.89 (1.05; 3.43)	1.76 (0.96; 3.24)
≥3	0.09 (−0.03; 0.20)	0.04 (−0.12; 0.20)	0.28 (0.12; 0.65)	0.29 (0.10; 0.78)	0.99 (0.55; 1.80)	0.91 (0.47; 1.75)
**Polypharmacy (vs. ≤4)**						
5–9	0.04 (−0.09; 0.18)	0.02 (−0.14; 0.18)	1.35 (0.56; 3.22)	1.74 (0.63; 4.79)	1.09 (0.56; 2.12)	1.09 (0.53; 2.21)
≥10	0.11 (−0.03; 0.25)	0.09 (−0.09; 0.28)	0.30 (0.11; 0.83)	0.52 (0.16; 1.76)	1.32 (0.66; 2.63)	1.36 (0.62; 2.96)

Abbreviations: CCI: Charlson’s Comorbidity Index; OR: odds ratio; CI: confidence intervals; GFI: Groningen Frailty Index; A&E: accident and emergency; * *p* < 0.05.

**Table 5:** Study 2 multivariate unadjusted and adjusted co-efficients and odds ratios for the change in health outcomes (HRQOL, A&E visits, functional impairment) between baseline and follow-up (T2b-T1b) for patients with an ADE compared to those without (non-ADE).

CPCR	EQ-5D-L3 (Follow-Up T2b- Baseline T1b) (N = 591)		Functional Decline (VES) (Follow-Up T2b- Baseline T1b) (N = 591)		A&E Visits (Follow-Up T2b- Baseline T1b) (N = 589)	
	Unadjusted Univariate Coef. (95% CI)	Adjusted Coef. (95% CI)	Unadjusted Univariate OR (95% CI)	Adjusted OR (95% CI)	Unadjusted Univariate OR (95% CI)	Adjusted OR (95% CI)
**ADE (vs. non-ADE)**	0.01 (−0.03; 0.04)	0.01 (−0.04; 0.05)	1.48 (0.82; 2.68)	1.35 (0.78; 2.34)	0.82 (0.53; 1.27)	0.70 (0.46; 1.08)
**Age**	0.00 (−0.00; 0.01)	0.00 (−0.00; 0.01)			1.00 (0.96; 1.03)	0.99 (0.95; 1.03)
**Male (vs. female)**	0.01 (−0.02; 0.04)	0.01 (−0.02; 0.05)	0.65 (0.44; 0.97)	0.70 (0.46; 1.05)	1.04 (0.76; 1.44)	1.00 (0.72; 1.38)
**CCI (vs. none)**						
1–2	−0.00 (−0.04; 0.03)	−0.01 (−0.04; 0.03)	0.95 (0.55; 1.62)	0.89 (0.47; 1.69)	1.10 (0.77; 1.57)	1.16 (0.90; 1.50)
≥3	−0.07 (−0.14; −0.00)	−0.07 (−0.15; 0.01)	1.51 (0.84; 2.73)	1.29 (0.67; 2.50)	0.65 (0.29; 1.49)	0.61 (0.30; 1.03)
**Polypharmacy (vs. ≤4)**						
5–9	0.01 (−0.03; 0.05)	0.01 (−0.04; 0.05)	1.05 (0.77; 1.42)	0.94 (0.69; 1.28)	1.14 (0.85; 1.54)	1.33 (0.93; 1.90)
≥10	0.01 (−0.03; 0.05)	0.00 (−0.07; 0.07)	1.72 (1.20; 2.48)	1.47 (0.83; 2.59)	1.31 (0.76; 2.26)	1.59 (0.95; 2.68)

Abbreviations: CCI: Charlson’s Comorbidity Index, OR: odds ratio, CI: confidence intervals, VES: Vulnerable Elderly Survey, A&E: accident and emergency.

## Data Availability

The datasets analysed during the current study are not publicly available, as they are subject to ongoing analysis by the authors, but are available from the corresponding author on reasonable request.

## Abbreviations

A&E	accident and emergency
ADE	adverse drug events
ADR	adverse drug reactions
CCI	Charlson Comorbidity Index
CI	confidence intervals
CPCR	Centre for Primary Care Research
GFI:	Groningen Frailty Index
GP	general practitioner
HRQOL	health-related quality of life
VES	The Vulnerable Elders Survey
